# Preparation of Polyvinyl Alcohol (PVA)-Based Composite Membranes Using Carboxyl-Type Boronic Acid Copolymers for Alkaline Diffusion Dialysis

**DOI:** 10.3390/polym12102360

**Published:** 2020-10-14

**Authors:** Lizhen Peng, Xiaonan Huang, Dandan Liu, Jibin Miao, Bin Wu, Ming Cao, Qianqian Ge, Bin Yang, Lifen Su, Ru Xia, Zhengzhi Zheng, Peng Chen, Jiasheng Qian

**Affiliations:** School of Chemistry & Chemical Engineering, Anhui University, Anhui Province Key Laboratory of Environment-friendly Polymer Materials, Hefei 230601, China; xiaozahn0824@163.com (L.P.); 19966505287@163.com (X.H.); 18756563397@163.com (D.L.); 13105@ahu.edu.cn (M.C.); gqq@mail.ustc.edu.cn (Q.G.); yangbin@ahu.edu.cn (B.Y.); ausulf@sina.com (L.S.); xiarucn@sina.com (R.X.); zzzhi@tom.com (Z.Z.); chpecp@126.com (P.C.)

**Keywords:** carboxyl-type boronic acid copolymers (CBACs), composite membranes, alkali recovery, diffusion dialysis (DD)

## Abstract

Carboxyl-type boronic acid copolymers (CBACs) were synthesized by a radical polymerization method and used for the preparation of polyvinyl alcohol (PVA)-based composite membranes via a solution mixture method. The as-prepared composite membranes exhibited a water uptake (W_R_) of 122.6–150.0%, an ion exchange capacity (IEC) of 0.0147–0.0518 mmol g^−1^, and excellent mechanical (elongation at break (E_b_) of 103.8–148.4%, tensile strength (TS) of 38.7–58.6 MPa) and thermal stability. The alkali resistances of the as-prepared membranes were tested by immersing the samples into 2 mol L^−1^ NaOH solutions at 25 °C for 60 h, and the results were encouraging: the mass loss and swelling degree of the as-prepared membranes were in the ranges of 1.9–5.9% and 222.6–241.9%, respectively. The separation performances of the as-prepared membranes were evaluated by the diffusion dialysis (DD) process with an NaOH/Na_2_WO_4_ mixture at room temperature. The results demonstrated that the dialysis coefficients of hydroxide (U_OH_) were in the range of 0.0147–0.0347 m h^−1^, and the separation factors (S) were in the range of 29.5–62.6. The introduced carboxyl groups from CBACs and the –OH groups from PVA were both deemed to play significant roles in the promotion of ion transport: the –COO^−^ groups formed negatively charged transport channels for Na^+^ by electrostatic attraction, and the –OH groups promoted the transport of OH^−^ via hydrogen bonding.

## 1. Introduction

The polymer blends are widely used in the fields of nanofiltration [1], membrane contactors [2], fuel cells [3,4], biorenewable resources [5], and capacitive deionization [6]. As a membrane separation technology that presents low energy consumption, low cost, and eco-friendliness, diffusion dialysis (DD) has exhibited broad application prospects in acid and basic wastewater treatment [7,8,9,10,11,12]. Compared with commercial applications of recovering acid via diffusion dialysis, studies of alkali recovery by diffusion dialysis are relatively limited. The key reason for this is the lack of suitable membranes, which should possess high OH^−^ coefficients and ion selectivity, satisfactory mechanical and thermal stability, and excellent alkali resistance [13,14]. The alkali resistance of anion exchange membrane is the key to wide application.

To obtain membranes applicable for alkali diffusion dialysis, many polymer materials have been investigated, including poly (2,6-dimethyl-1,4-phenyleneoxide) (SPPO) [14,15], polyvinyl alcohol (PVA) [16,17,18,19], polyvinylidene fluoride (PVDF) [20,21,22,23], chlorosulfonated polyethylene (CSM) [24], and so on. Among these polymer matrix materials, PVA possesses good hydrophilicity and membrane-forming abilities and has drawn researchers’ attentions.

For PVA-based membranes, it is necessary to introduce ion exchange groups and cross-linked structures to endow the membranes with ion exchange abilities and enhance their sustainability during operation. Therefore, many researchers have focused on this field, and certain achievements have been obtained. Liu et al. [17] mixed poly (p-styrene sulfonate) with PVA to introduce sodium sulfonate groups, and a series of cation exchange membranes (CEMs) were obtained. The as-prepared PSSNa/PVA-PSf composite membrane possessed improved water flux, efficiency of anionic dye removal, and fouling resistance to bovine serum albumin (BSA) aqueous solution. The authors considered that the improved membrane permeability and solute selectivity was due to the incorporation of PSSNa, which resulted in increased membrane pore size, surface hydrophilicity, and negative charge. Dai et al. [18] prepared PVA-based membranes by directly blending 3-mercaptopropyltriethoxysilane and benzaldehyde disulfonic acid disodium salt with PVA solution. The –CHO and –Si(OC_2_H_5_)_3_ groups can easily cross-link with –OH groups through acetal condensation and sol-gel reactions, endowing the membranes with exchangeable groups and cross-linked structures. The as-prepared membranes were applied to the DD process for alkali recovery, with the –SO_3_^−^ and –OH groups anticipated to enhance NaOH permeability, and thus, the DD performance improved: the dialysis coefficients of NaOH (U_OH_) in NaOH/NaAlO_2_ solution were in the range of 2.8–23.4 mm h^−1^ along with the separation factors (S) ranging between 4.6 and 20.0. Wu et al. [19] prepared a kind of multiple-silicon copolymer using maleic anhydride and methacryloxypropyl trimethoxy silane as monomers, which contained a large amount of siloxane and anhydride groups. The copolymer was used to blend with PVA solution, and a series of hybrid membranes were obtained by in situ sol-gel reaction. The U_OH_ were higher than those of conventional ion exchange membranes at both 20 °C and 40 °C (the U_OH_ values were in the range of 0.0095–0.0123 m/h at 20 °C and 0.0155–0.0208 m/h at 40 °C). The double –COOH structure was considered to play important roles during ion transport. A green method for the preparation of PVA-based cation exchange hybrid membranes has been introduced by Hao et al. [25] through the use of 3-trihydroxysilyl-1-propanesulfonic acid (THOPS). THOPS contains both cation exchange groups (–SO_3_H) and Si(OH)_3_ groups, which can directly cross-link with PVA by the condensation reaction between Si–OH and PVA–OH groups. The obtained membranes exhibited potential application in alkaline diffusion dialysis, with U_OH_ values in the range of 0.011–0.022 m h^−1^ and S values in the range of 11.6–20.6.

In our previous work, Liu et al. [26] prepared sulfonic acid-type boronic acid copolymers (BACs) and confirmed the potential application of BACs in the preparation of PVA-based composite membranes for alkaline diffusion dialysis. The as-prepared membranes exhibited encouraging separation performance, with optimal OH^−^ dialysis coefficient (U_OH_) and separation factor (S) values of 0.0079–0.0150 m h^−1^ and 53.2, respectively. To extend the application of BACs and determine the relationship between BACs and the separation performance of composite membranes, we synthesized a novel kind of carboxyl-type BAC and introduced it to prepare PVA-based composite membranes. The monomer ratio of BACs was evaluated by the results of the diffusion dialysis test, and the as-prepared membranes exhibited relatively higher U_OH_ and S values than those of a previous report, which would be due to the similar molecular chain structures and good compatibility between the PVA and the carboxyl-type BACs. The alkaline resistance of the membranes has been effectively improved.

## 2. Materials and Methods

### 2.1. Materials

PVA (purity greater than 99%), ammonium persulphate ((NH_4_)_2_S_2_O_8_), sodium acrylate (SA), sodium hydrogen sulfite (NaHSO_3_), Na_2_WO_4_, NaOH, and HCl of analytical purity were purchased from Sinopharm Chemical Reagent Co., Ltd. (Shanghai, China). Additionally, 3-acrylamido phenylboronic acid (AAPBA) of analytical purity was purchased from Boron Nobel Technology Co., Ltd. (Beijing, China). A 5% solution of PVA was formed by dissolving the polymer into deionized water as described in previous work [24]. The mixture of NaOH (1.0 mol L^−1^)/Na_2_WO_4_ (0.10 mol L^−1^) was prepared fresh [13], and deionized water was used during the experiment.

### 2.2. Synthesis of Boronic Acid Copolymers

**Scheme 1:**
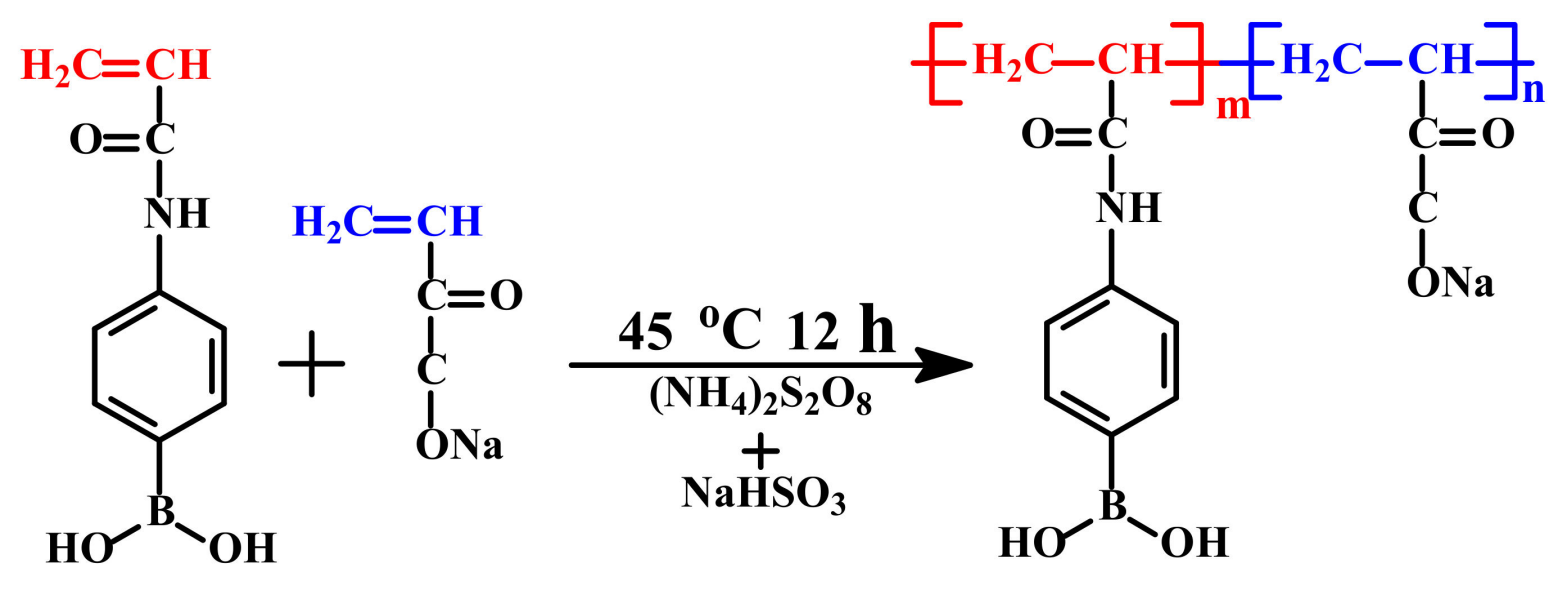
Synthesis process of boronic acid copolymers (BACs).

Similar with our previous work [26], the typical synthesis process of BACs was as follows: 0.94 g SA and 0.191 g AAPBA were dissolved in 60 mL deionized water and stirred by magnetic force to form a transparent solution. At this time, the pH value was measured as 7. Then, 15.2 mL NaOH (1 g L^−1^) was added to the solution above at stirring until the solution pH was adjusted to 8. Next, a mixture of 4.5 mg of SHS and 13.6 mg of APS was added into the above-mentioned solution under continuous stirring at room temperature. The transparent solution was heated to 45 °C and maintained for 12 h under vigorous stirring. Finally, an aqueous solution of BACs was obtained, and the BAC was partly precipitated and washed with ethanol several times. The obtained BACs were stored in glass desiccators for characterization, and the reaction process was displayed in Scheme 1.

### 2.3. Preparation of PVA-Based Composite Cationic Membranes

The obtained BACs aqueous solution was blended with 40 mL of 5% PVA solution under vigorous stirring with various mass ratios (BACs to PVA): 0%, 0.5%, 1%, 2%, and 4%. The pH of the mixture was maintained at 8–9, and the reaction time was 30 min at 25 °C. The obtained solutions were cast onto a clean glass plate (20 cm × 20 cm) and dried in air at 25 °C. The as-prepared membranes were peeled off and heated from 60 to 100 °C with a heating rate of 10 °C h^−1^ after the solvent had evaporated completely. These treatment conditions were in accordance with our previous work [25], and the as-prepared membranes were marked as 0%, 0.5%, 1%, 2%, and 4%, based on the mass ratio of BAC to PVA. The viscosity of the casting solution increased obviously when the dosage of BACs reached 6% and it is difficult to form a membrane; therefore, the upper limit dosage of BACs was set at 4%.

### 2.4. Characterizations and Diffusion Dialysis (DD) Testing

The Fourier transform infrared spectroscopy (FTIR) of BACs was obtained on a Nicolet iS10 spectrometer and the scanning range was set from 400 cm^−1^ to 4000 cm^−1^. The ^1^H-NMR spectrum of BACs was obtained by a Bruker NMR spectrometer (Advance II 400 MHz, Bruker, Germany). The thermal stabilities of the as-prepared membranes were tested by a Shimadzu TGA-50H analyzer (Tokyo, Japan) under a nitrogen atmosphere at a heating rate of 10 °C min^−1^ from room temperature to 800 °C. The mechanical properties of the as-prepared membranes were measured by a microcomputer control electronic universal testing machine (Instron 5967, Instron (Shanghai) Test Equipment Trading Co., Ltd., Shanghai, China) at 25 °C, and the samples were cut into a dumbbell shape before testing. The tensile rate was set at 25 mm min^−1^, and the initial gauge length was 25 mm. The microstructure of the membranes was observed by microscopic scanning electron microscopy (S-4800, Hitachi Company, Tokyo, Japan) after gold coating treatment. The water uptake (W_R_), ion exchange capacities (IECs), and alkali resistance of the membrane samples were tested by the methods that have been described in our previous works, and two parallel samples were tested synchronously [27,28].

In a typical DD test, the membrane samples were first immersed in the NaOH/Na_2_WO_4_ mixture for 2 h and then fixed between two separated compartments. The NaOH/Na_2_WO_4_ mixture and deionized water were placed into the two compartments. The effective areas were 6 cm^2^, and the liquid volume in each compartment was 100 mL. Both solutions in each compartment were stirred at the same rate to reduce the influence of concentration polarization. The testing time was controlled at 1 h, which was in line with our previous work [15,29]. Next, the mixture and the diffusate were removed from both sides. The concentration of OH^−^ was determined by the HCl titration method, while the concentration of WO_4_^2−^ was measured by thiocyanate spectrophotometry [30]. The separation factor (S) was the ratio of the dialysis coefficient (*U*) of OH^−^ and WO_4_^2−^. The dialysis coefficient could be calculated by the following formula:

(1) $$U=\frac{M}{At\Delta C}$$

where *M* is the molar amount of material transfer (mol), *A* is the effective area (m^2^), *t* is the experimental time (h), and Δ*C* is the average logarithm of the concentration of two compartments (mol m^−3^). The calculated formula was as follows:

(2) $$\Delta C=\frac{C_{f}^{0}-(C_{f}^{t}-C_{d}^{t})}{\ln[C_{f}^{0}/(C_{f}^{t}-C_{d}^{t})]}$$

where $C_{f}^{0}$ and $C_{f}^{t}$ are the ion concentrations of the diffusate at time 0 and *t*, respectively, while $C_{d}^{t}$ is the ion concentration of the feed solution at time *t*.

## 3. Results

### 3.1. Structure Characterization of BACs

**Figure 1:**
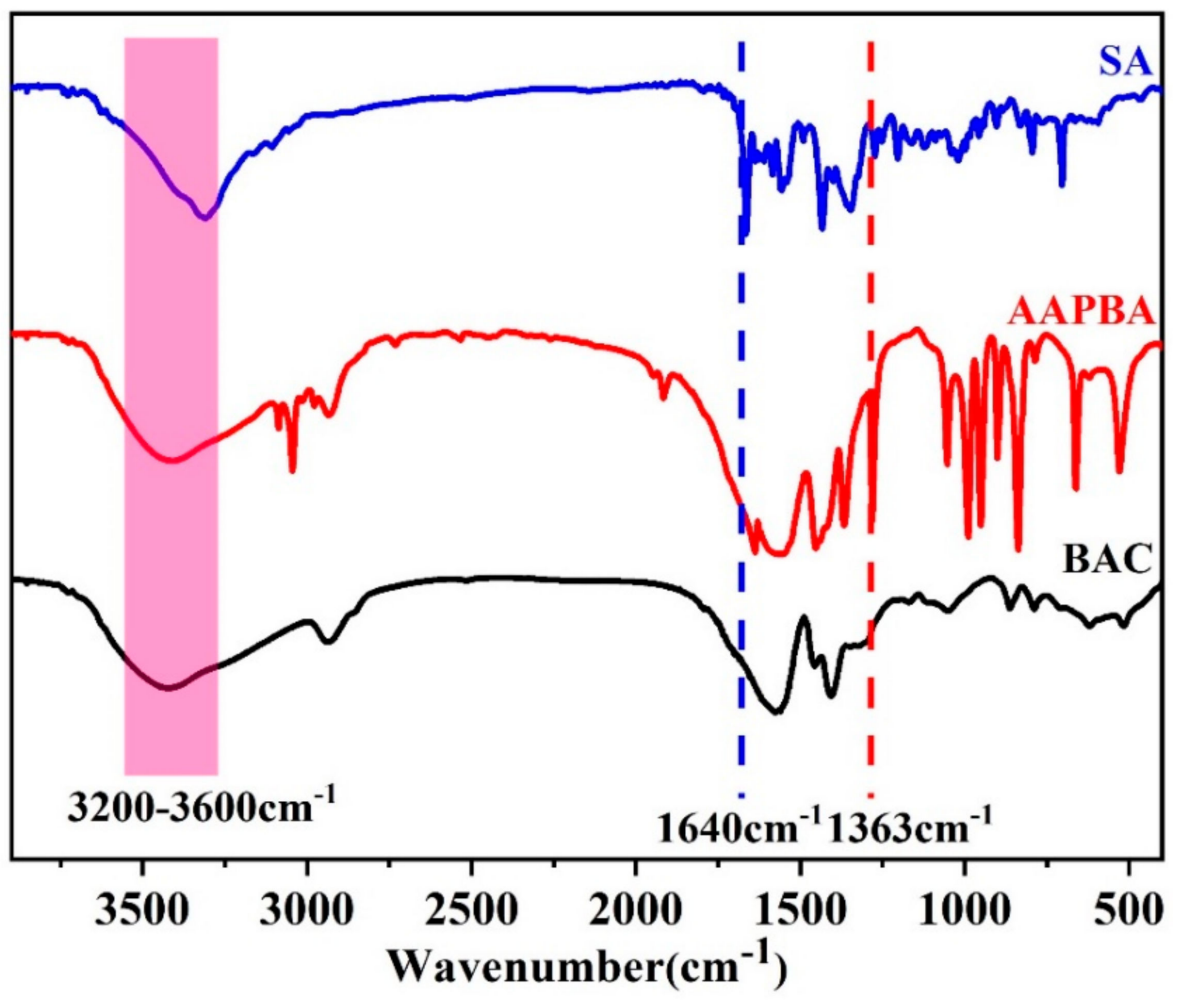
Fourier transform infrared spectroscopy (FTIR) of the BACs and monomers.

**Figure 2:**
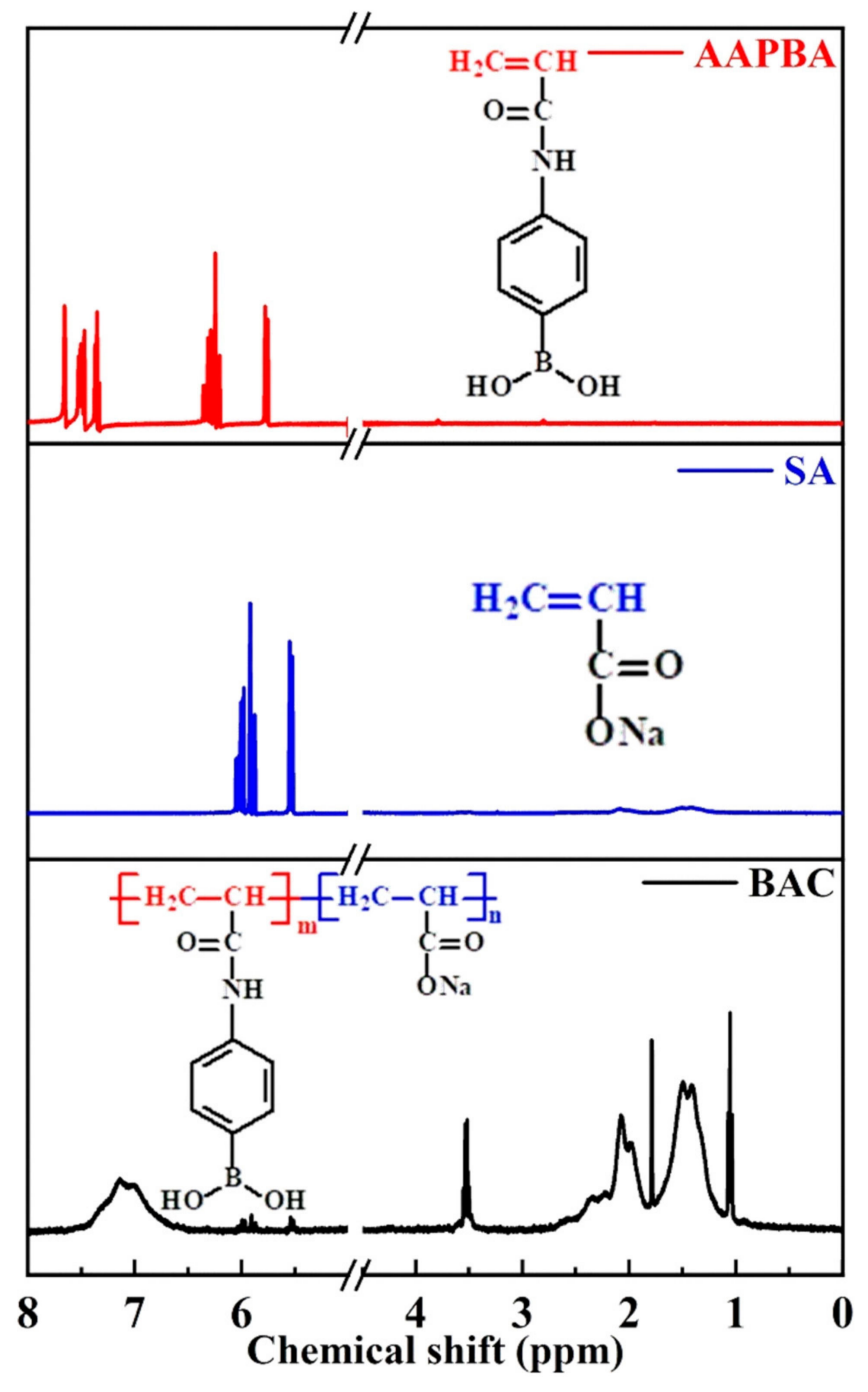
^1^H-NMR spectrum of the 3-acrylamido phenylboronic acid (AAPBA), sodium acrylate (SA) and BACs in D_2_O.

FTIR and ^1^H-NMR analyses were performed to confirm the structure of the synthesized BACs, and the results are shown in Figure 1 and Figure 2, respectively. The FTIR spectra of the BACs and monomers are shown in Figure 1. The tensile vibration absorption peak of C=C at 1640 cm^−1^ disappeared in the FTIR spectrum of BACs, confirming the successful polymerization between AAPBA and SA. The broad peak in the range of 3200–3600 cm^−1^ corresponded to the stretching vibrations of –OH groups, which indicated that the polymer contained a large amount of hydroxyl groups. The tensile vibration absorption peak of COO^−^ at 1363 cm^−1^ indicated that the carboxyl group was successfully bonded to AAPBA.

The ^1^HNMR spectrum of the as-synthesized BACs in D_2_O was shown in Figure 2. It could be observed from the figure that δ = 3.6 ppm and 1.1 ppm were the methylene and methyl peaks of the solvent ethanol, respectively. As the ^1^HNMR of the polymer was not as clear as the monomer, some peaks were attributed mainly to the position of the peaks. The broad peak at δ = 6.5–8.0 ppm was attributed to protons on the benzene ring, and the strong peak at 4.8 ppm was from the solvent hydrazine. The proton chemical shift of –CH=CH_2_ in the monomer was 5–6 ppm, and this signal was absent in the polymer, with concomitant growth of signals at 1.2–3.0 ppm, which suggests that the vinyl protons disappear in the polymer. The proton chemical shifts of –CH and –CH_2_ overlap each other, and the tensile vibration peak corresponding to C=C in the infrared spectrum disappeared. Based on the above results, we conclude that BACs were successfully synthesized.

### 3.2. IECs and W_R_ of As-Prepared Membranes

The W_R_ and IEC results of the as-prepared membranes are shown in Table 1. The W_R_ values were in the range of 122.6–150.0% and all the composite membranes exhibited higher hydrophilicity than that of the blank membrane sample, which indicated that the involvement of BACs promoted the adsorption of water. This outcome could be explained regarding two aspects: First, the –COO^−^ groups possessed higher hydrophilicity than –OH groups [27], and thus, the W_R_ of composite membranes increased with increasing BACs dosage; and second, the cross-linked reaction between the –OH and –B(OH)_2_ groups could increase the density of the composite membranes, which would prevent the membranes from adsorbing water molecules. Therefore, the W_R_ of the as-prepared membranes first markedly increased and then slightly decreased as the dosage of BACs increased to 4%. The W_R_ of the composite membranes was in a moderate range compared with that observed in previous research [26], making it suitable for alkaline DD.

The IECs of the as-prepared membranes are shown in Table 1. Compared with those in previous reports [15,26,28,29], the IECs of the membranes in this work were relatively lower. As the IECs of the as-prepared membranes were mainly derived from the number of –COO^−^ groups, the IEC values increased gradually from 0.015 to 0.052 mmol g^−1^ with increasing BACs dosage. This result also confirmed the successful cross-linking reactions between PVA and BACs. Table 2 sums up the IEC, U_OH_, and S values reported in previous studies on membranes. It can be seen from the data that the higher U_OH_ and S values were obtained at lower IEC in our work. Therefore, the membrane structure plays an important role in membrane performance.

### 3.3. Alkali Resistance Testing Results of As-Prepared Membranes

**Figure 3:**
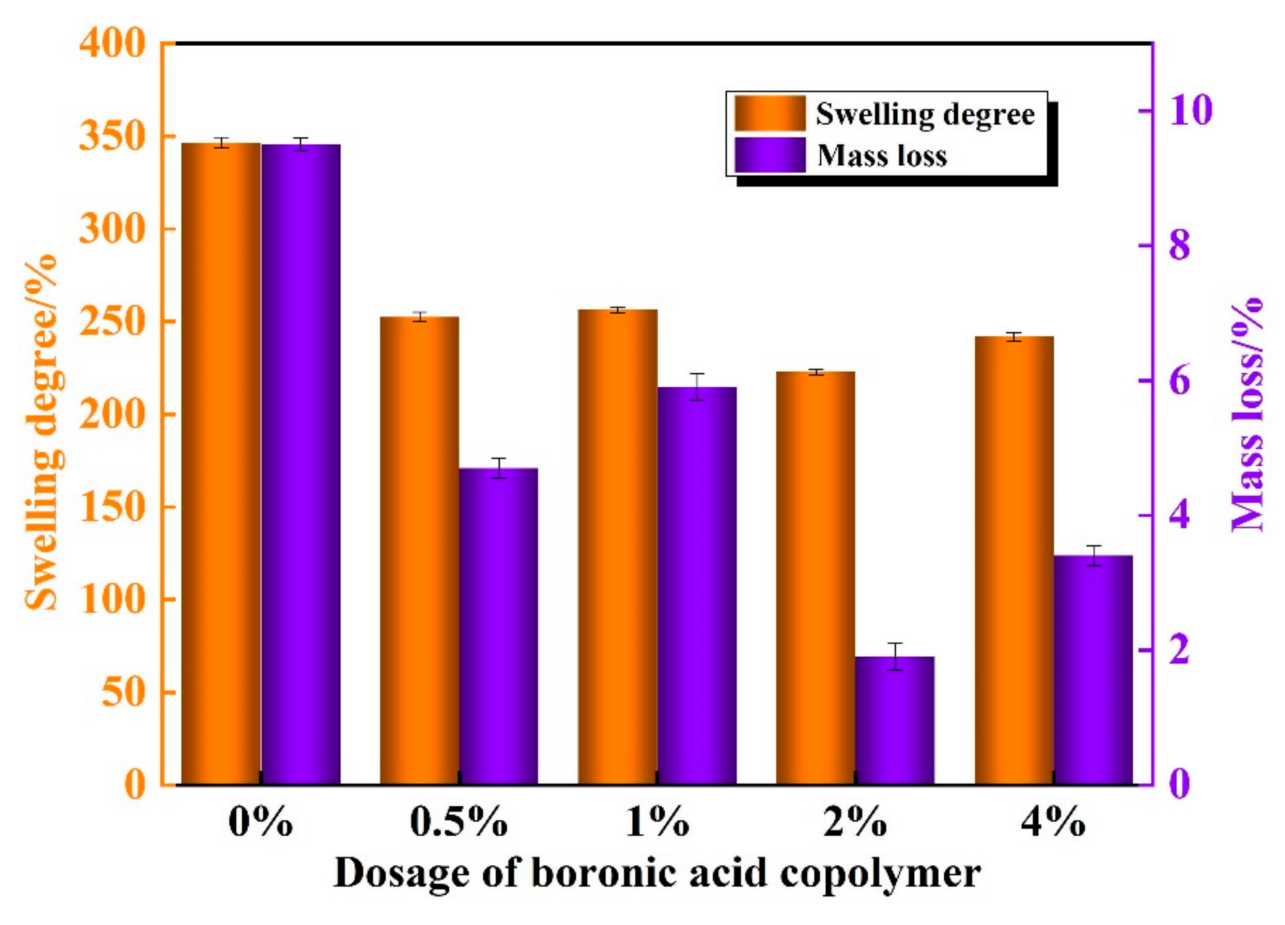
Alkali resistance testing results of as-prepared membranes.

The alkali resistance of the as-prepared membranes was represented by the swelling degree and mass loss results, which are shown in Figure 3. The composite membranes exhibited a lower swelling degree and mass loss than those of the pure PVA membrane, which indicated that the incorporation of BACs into the PVA matrix was advantageous for promoting the alkali resistance of the composite membranes. The swelling degrees of composite membranes were in the range of 222–250% and tended to decrease first and then slightly increase as the dosage of BACs increased to 4%. The reasons might be as follows: the swelling resistance of the composite membranes was enhanced compared with that of pure PVA when the dosage of BACs was less than 4%, which was mainly due to the increased density as the dosage of BACs increased. When the dosage of BACs was 4%, the slightly increased tendency of the swelling degree was mainly due to the increased content of the –COO^−^ groups, which were more hydrophilic than the –OH groups.

The mass loss of the composite membranes was in the range of 1.9–5.9%, which was far lower than that of the pure PVA membrane (9.7%). Considering that the weight loss of the composite membranes was primarily due to the dissolution of dissociated PVA chains, the results revealed that the cross-linked structure between the –OH and –B(OH)_2_ groups increased the density of the composite membranes and prevented the attack of OH^−^ on the PVA matrix. The composite membranes exhibited excellent alkali resistance at room temperature and were suitable for alkaline diffusion dialysis application [31,32]. The composite membranes showed optimal alkali resistance when the dosage of BACs was 2%, with a swelling degree of 222.6% and a mass loss of 1.9%.

### 3.4. Mechanical Properties of As-Prepared Membranes

The mechanical properties of the as-prepared membranes, including tensile strength (TS) and elongation at break (E_b_), are shown in Table 3. The TS and E_b_ of the as-prepared membranes were in the range of 38.7–58.6 MPa and 103.8–148.4%, respectively. Compared with that of the blank sample, the TS values of the composite membranes indicated trends of first decreasing and then increasing. The cross-linking reaction between –B(OH)_2_ groups from BAC and the hydroxyl groups of the PVA main chain could cause rearrangement of the PVA chains, which destroyed the ordered arrangement of the original polymer chains and resulted in a decrease in the strength and flexibility of the composite membranes. As the dosage of BACs increased, the number of cross-linking points between PVA and BACs increased, and the molecular chains became less prone to moving. Thus, the TS values of the membranes increased markedly. The E_b_ values of the as-prepared membranes were significantly reduced when the number of BACs was less than 4%, which was due to the restricted motion of the PVA chains with increasing cross-linking degree. When the dosage of BACs increased to 4%, the obvious increase in the E_b_ value was attributed to the increasing number of tangling points between the PVA and BACs, as well as the number of hydrogen bonds between the –B(OH) groups of PVA [29].

### 3.5. Thermal Stability Properties of As-Prepared Membranes

**Figure 4:**
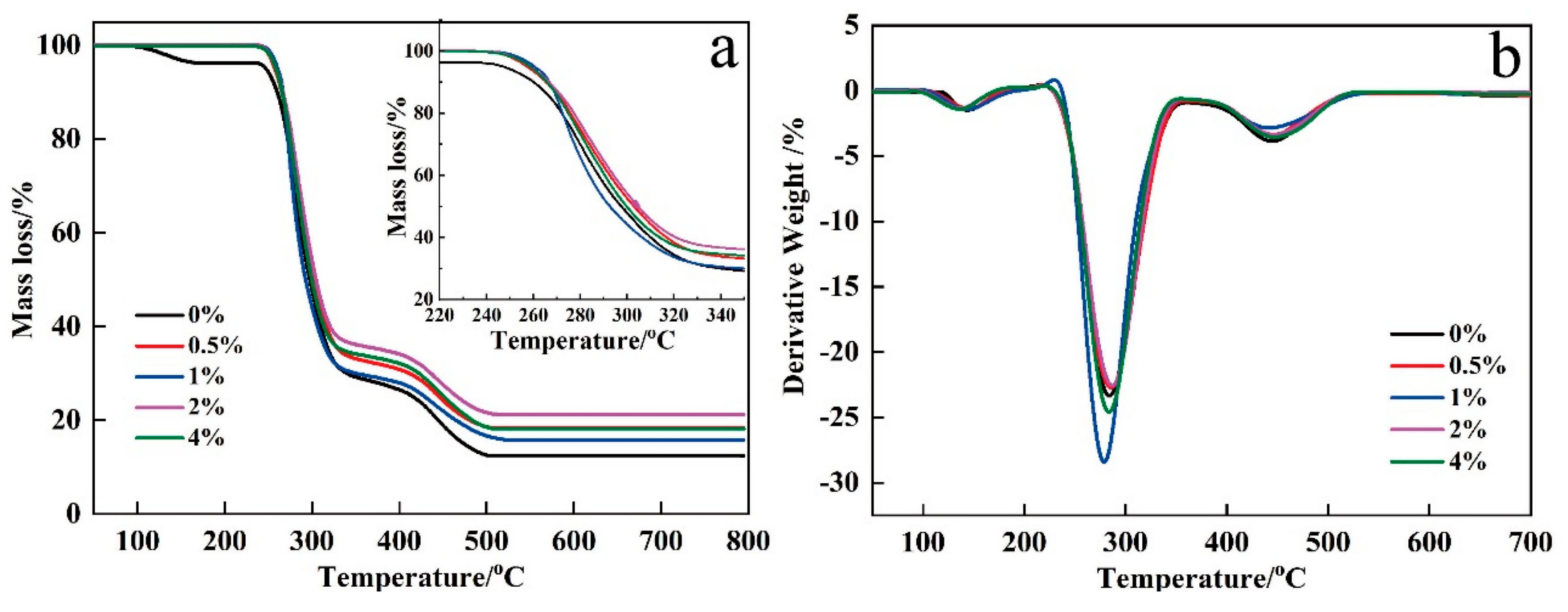
Thermogravimetric (TG) (**a**) and differential thermogravimetric (DTG) (**b**) curves of the as-prepared membranes.

The thermogravimetric (TG) and differential thermogravimetric (DTG) curves of the as-prepared membranes are shown in Figure 4 and Table 4, from which three mass loss peaks of the composite samples could be observed. The first peak before 200 °C corresponded to the loss of adsorbed water. The second peak in the range of 200–300 °C corresponded to the decomposing of OH^−^ and –COO^−^ groups [27], from which a complicated tendency could be observed, which was due to the forming of a cross-linked interaction between –OH and –B(OH)_2_ groups. The results showed that increasing amounts of BAC had little effect on the thermal stability of the functional groups. The OH^−^ and –COO^−^ groups tended to degrade easily due to the rearrangement of the PVA molecular chains. The third weight loss peak between 400 and 500 °C corresponded to the decomposing of the PVA backbone. The residual mass of the composite membranes was obviously higher than that of pure PVA, which indicated that the enhanced entanglement between the PVA molecules and BAC was also advantageous for the thermal stability of the membrane backbones. In short, the presence of BACs exhibited slight effects on the thermal stability of the OH^−^ and –COO^−^ groups, while the stability of the carbon chains was obviously promoted.

### 3.6. Microstructures of As-Prepared Membranes

**Figure 5:**
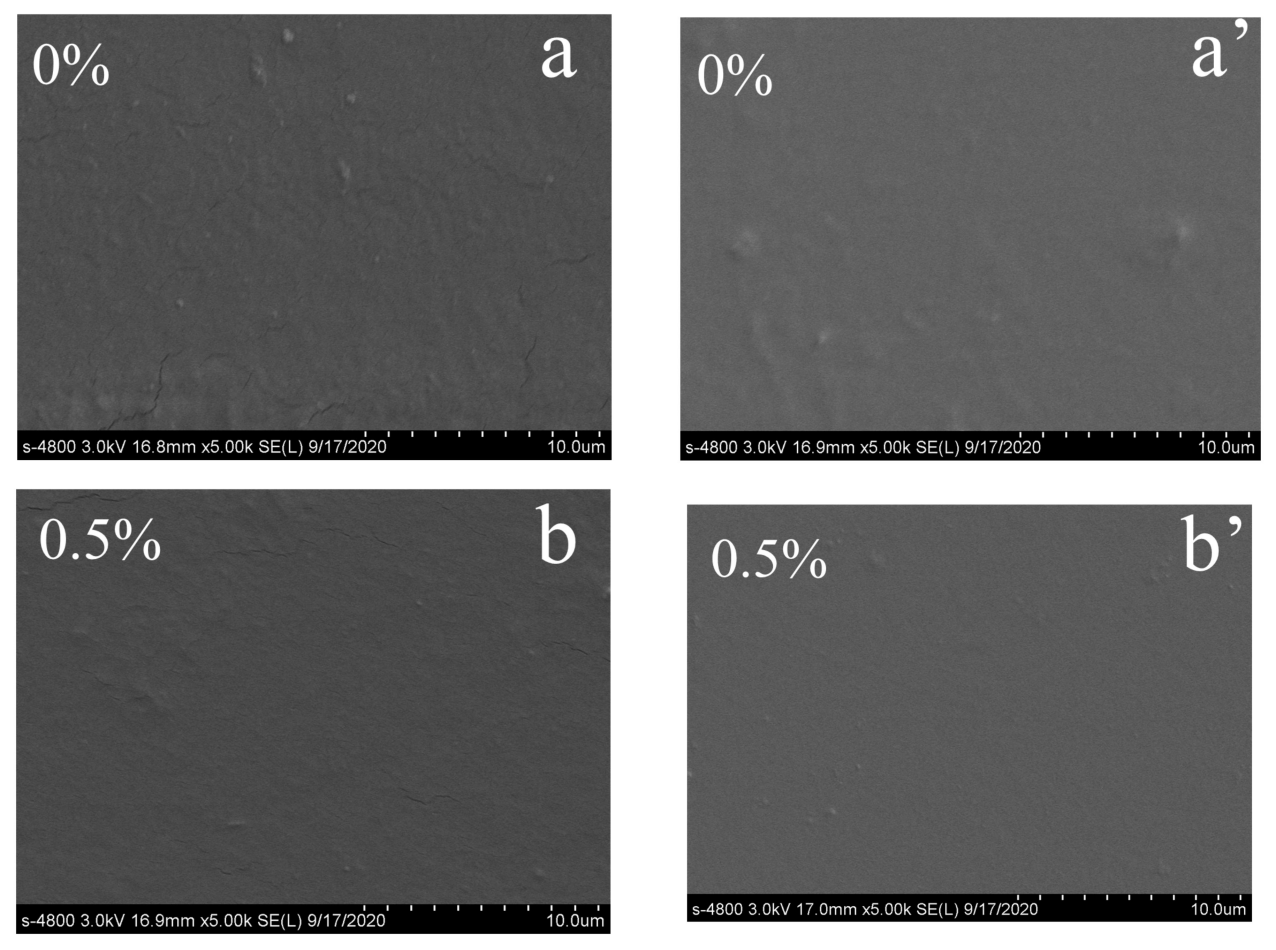

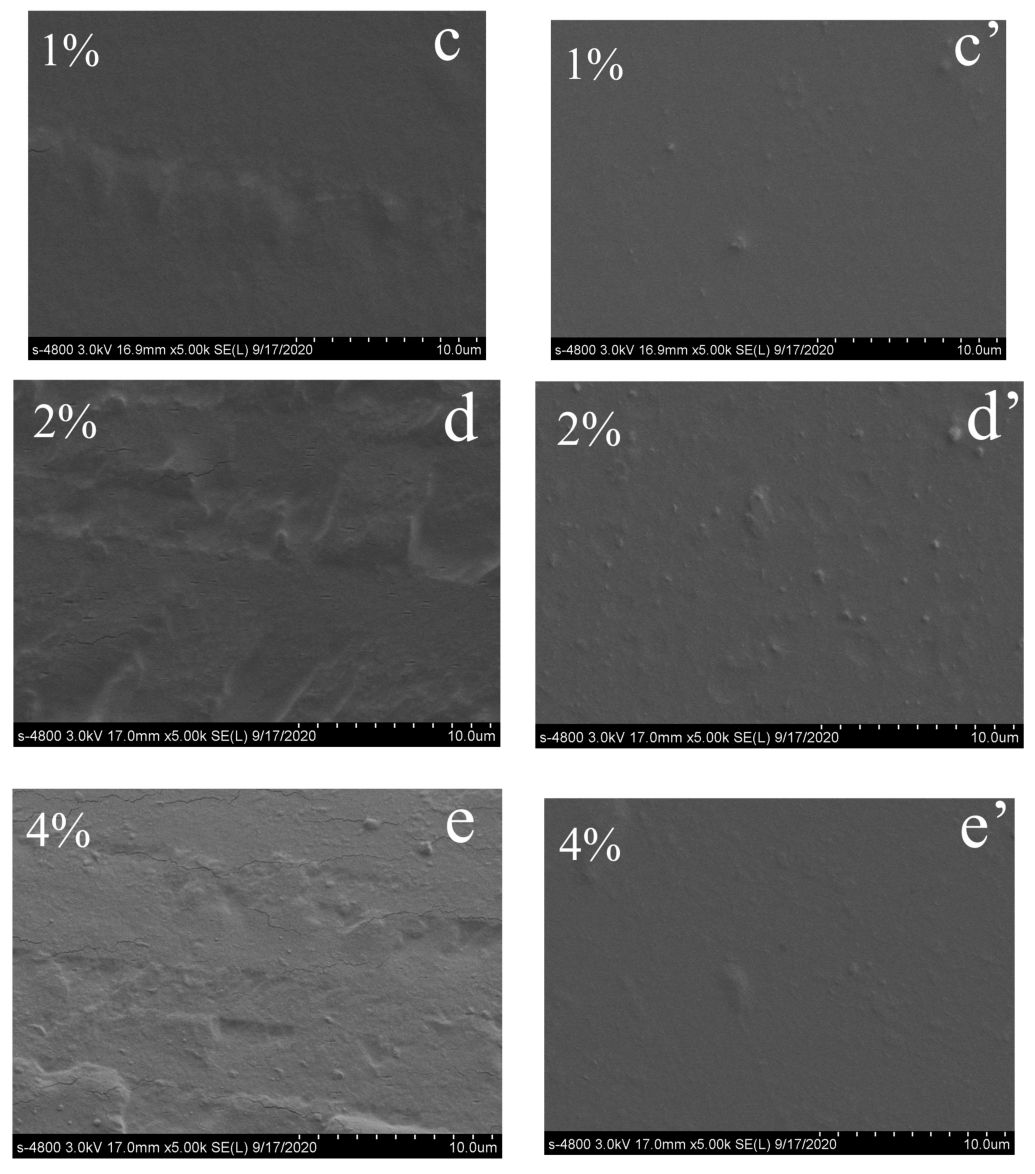
Cross-sectional (**a**–**e**) and surfaces (**a’**–**e’**) scanning electron microscopy (SEM) images of the as-prepared membranes with different mass ratios of BACs.

The cross-section (a–e) and surface (a’–e’) SEM images of the as-prepared membrane with different mass ratios of BACs are exhibited in Figure 5. With the increase of graphene content, due to its good compatibility, no obvious phase separation was observed on the surface. However, when the mass fraction reached 4%, phase separation appeared on the cross section. We tried to increase the dosage of BACs (e.g., 6%), but there was high viscosity resulting in the heterogeneity of the nanocomposites; the solution cannot form a membrane. These membranes exhibited smooth, uniform and dense cross-sectional structures when the dosage of BACs was less than 4%. The results confirmed the compatibility between the PVA matrix and BACs, which was due to the forming of hydrogen bonds between the –OH and –B(OH)_2_ groups, as well as the structural comparability between PVA and BACs. However, the composite membrane revealed obvious phase separation when the dosage of BACs was 4%. The results indicated that excessive dosage of BACs had an obvious effect on the microscopic structure of the composite membranes, and thus, the membrane formation process became more difficult. Considering the microstructure of the as-prepared membranes, the dosage of BACs should be controlled to less than 4%, and then, composite membranes with dense, uniform microstructures can be obtained.

### 3.7. Results of the DD Test

**Figure 6:**
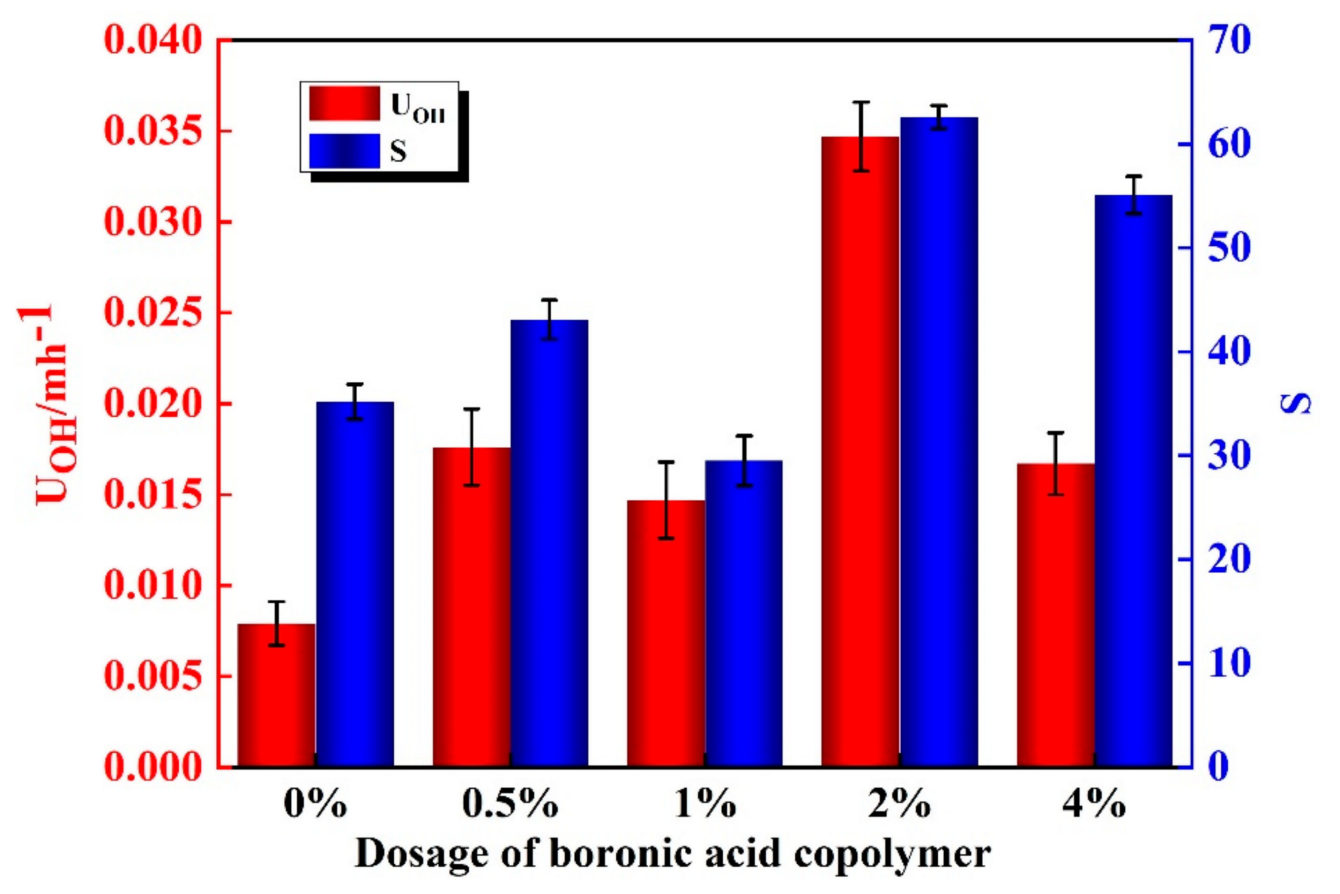
Diffusion dialysis (DD) results of as-prepared membranes.

The dialysis coefficients of OH^−^ (U_OH_) and the separation factors (S) of the as-prepared membranes are shown in Figure 6. The U_OH_ of the membranes was in the range of 0.0147 to 0.0347 m h^−1^, which was higher than that in most existing studies [18,25,31]. The results indicated that the presence of BACs in the PVA was advantageous to the enhancement of OH^−^ flux, while the influence of BACs introduction on the U_OH_ of the membrane was complicated. When the BACs dosage was 2%, the U_OH_ of the membrane was the highest, and the possible transfer mechanism was as follows: When the amount of BACs was low, the formation of the cross−linked structure was imperfect. The ion flux of the membrane mainly depended on ion permeation, and the transport of OH^−^ and Na^+^ mainly obeyed free diffusion [13], so the flux of the membrane was low; when the amount of BACs was 2%, the U_OH_ of the membrane was the highest, indicating that the suitable introduction of BACs could simultaneously promote ion flux and selectivity. The transport mechanism of OH^−^ and Na^+^ could be concluded as follows: –COO^−^ groups from BACs formed the main transport channels of Na^+^ via electrostatic attraction; OH^−^ was forced through the membrane via the electroneutrality principle; and thus, –OH groups in the PVA backbone established an assisted transport channel of OH^−^ through hydrogen bonds [29].

**Figure 7:**
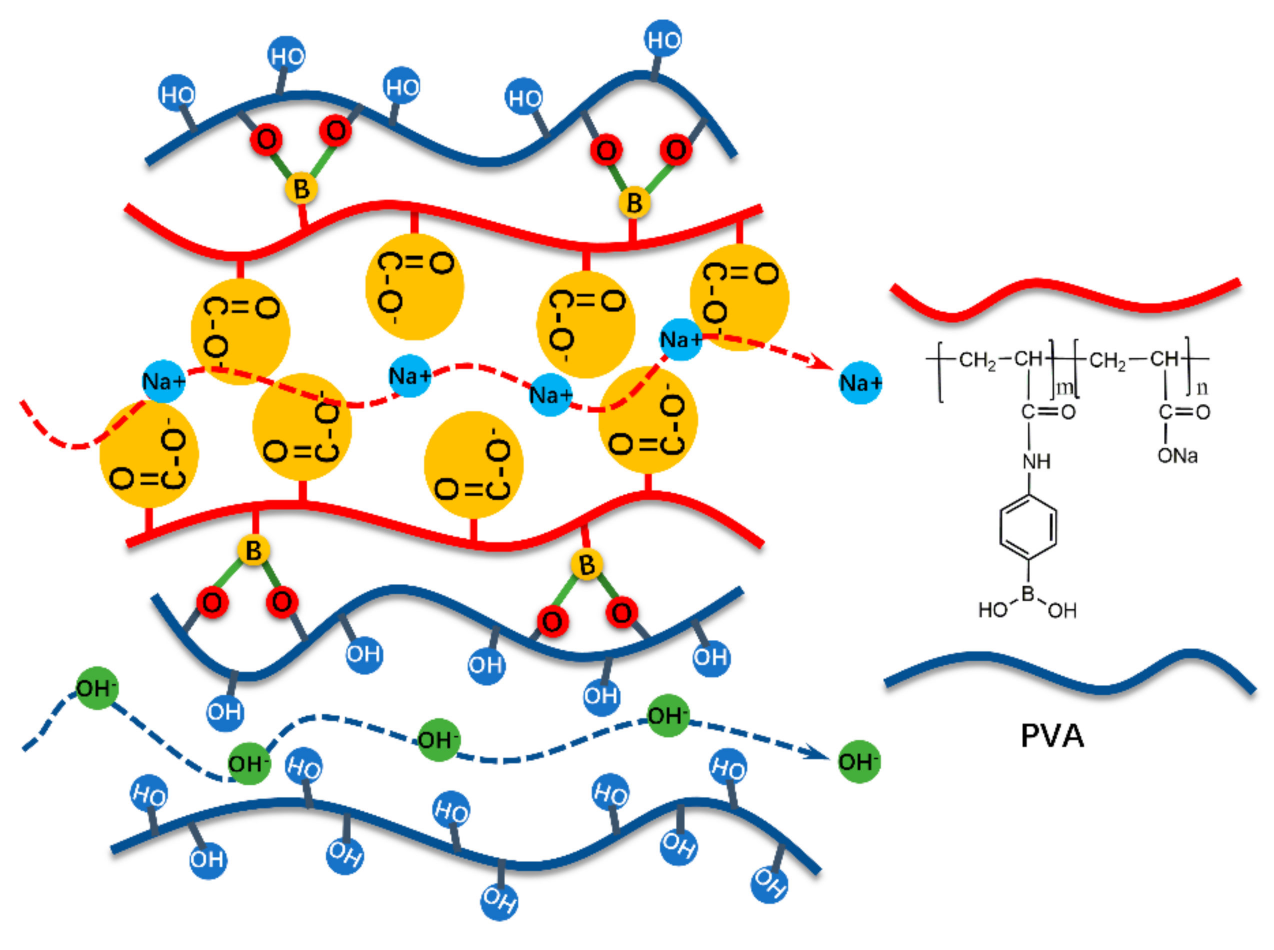
Proposed transport process of Na^+^ and OH^−^.

The proposed mechanism of ions is shown in Figure 7. Both the main transport channels and the assisted channels synergistically promoted the transport of OH^−^ and Na^+^. Thus, the U_OH_ decreased markedly as the dosage of BACs increased to 4%; the cross-linking between –B(OH)_2_ and PVA increased the density of the membranes, and the number of free –OH groups decreased accordingly as the BACs increased. Therefore, the separation performance of the composite membrane visibly decreased when the dosage of BAC was 4%. In summary, when the amount of BACs was 2%, the composite membrane exhibited optimal separation performance: the OH^−^ dialysis coefficient (U_OH_) was 0.0347 m h^−1^, and the separation factor (S) was 62.6.

To further evaluate the separation performance of membranes in this research, we summarized the related reports and the results are listed in Table 5. The conclusions could be obtained as follows:

(1) Hydrophilic polymer (such as PVA) possessed higher U_OH_ than that of hydrophobic ones (such as CSM and PVDF), while ion selectivity was the opposite. The results showed that the natural properties of the polymer matrix had significant effects on the separation performance of the membranes.

(2) Membranes in this research exhibited higher U_OH_ and S than those of the references, which indicated that involvement of BACs could be one of the choices to solve the “tradeoff effects” between ion dialysis coefficients and selectivity in the DD separation process.

## 4. Conclusions

A series of composite membranes were prepared by the blending-casting method, and the separation performance of the as-prepared membranes was evaluated by the recovery of alkali via diffusion dialysis. The as-prepared membranes possessed a moderate W_R_ (122.6–150.0%), low IECs (0.015–0.052 mmol g^−1^), and outstanding alkali resistance (swelling degree was in the range of 222.6–241.9% and mass loss was in the range of 1.9–5.9%), which indicated that the composite membranes were suitable for application in alkaline diffusion dialysis processes. All the composite membranes exhibited acceptable mechanical and thermal stability and uniform microscopic morphologies, which confirmed the good compatibility between the PVA and the BACs. The composite membrane possessed optimal separation performance when the dosage of BAC was 2%, with U_OH_ and S values of 0.0347 m h^−1^ and 62.6, respectively. Both the –COO^−^ groups from the BACs and –OH groups from the PVA were considered to play synergistic roles in promoting the transport of OH^−^ and Na^+^. This work provides a significant idea for further synthesis of anion exchange membranes for alkali recovery applications.

## Figures and Tables

**Table 1:** IECs, W_R_, and thickness of the as-prepared membranes.

BAC Dosage	W_R_ (%)	IEC (mmol/g)	Thickness (µm)
0%	92.2 ± 1.2	/	51 ± 2
0.5%	122.7 ± 0.9	0.015	77 ± 1
1%	129.7 ± 0.9	0.026	86 ± 1
2%	150.0 ± 0.8	0.038	73 ± 2
4%	137.5 ± 0.7	0.052	85 ± 1

**Table 2:** IEC, U_OH_, and separation factors (S) of membranes in previous literatures.

Membranes	IEC/mmol g^−1^	U_OH_/m h^−1^	S	References
PVA-P(AA-co-γ-MPS)	0.76–0.9	0.01–0.042	25.5–95.7	[10]
PVA-BADSANa-MPTES	0.98–1.36	0.0028–0.0234	4.6–20.0	[18]
PVA-P(AA-co-γ-MPS)	1.39–1.78	0.0095–0.0123	28.4–54.4	[19]
PVA-P(THOPS-Na)	0.7–1.56	0.011–0.022	11.6–20.6	[25]
PVA-P(AAPBA-AMPS)	0.79–1.17	0.008–0.015	15–55	[26]
PVA-P(SAS-co-VTMS)	0.14–0.45	0.0065–0.0086	24.4–51.6	[27]
PVA/SPPO/SiO_2_	0.44–1.34	0.007–0.013	12.5–181	[30]
PVA-(MS-HPW)	0.015–0.03	0.00767–0.0115	21.5–88.8	[31]
PVA-BAC	0.0147–0.0518	0.0147–0.0347	29.5–62.6	This Work

**Table 3:** Mechanical properties of the as-prepared membranes.

BACs Dosage	TS (MPa)	E_b_ (%)
0%	45.1 ± 2.4	131.3 ± 6.9
0.5%	38.7 ± 2.0	103.8 ± 3.0
1%	42.2 ± 2.6	108.1 ± 7.3
2%	58.6 ± 2.4	112.2 ± 3.6
4%	52.6 ± 2.3	148.4 ± 2.9

**Table 4:** IDT a and Td b values of the as-prepared membranes.

Dosage of Boronic Acid Copolymer	0%	0.5%	1%	2%	4%
IDT ^a^ (°C)	209	219	220	238	229
Td ^b^ (°C)	247	257	260	259	258

^a^ IDT is the initial decomposition temperature determined from thermo gravimetric analysis (TGA) thermograms. ^b^ Thermal degradation temperature (Td) is defined as the temperature at which the weight loss is 5% in the TGA thermograms.

**Table 5:** Separation performance comparisons (U_OH_ and S) of membranes in this research and previously reports.

Membrane Matrix	Separation System	U_OH_/m·h^−1^ (25 °C)	S (25 °C)	Ref.
PVA	NaOH/Na_2_WO_4_	0.011–0.022	11.6–20.6	[26]
PVA		0.0079–0.0150	26.6–53.2	[27]
PVA		0.0065–0.0086	24.4–51.6	[28]
SPPO		0.0035–0.0061	31.6–37.5	[29]
SPPO		0.002–0.0038	98–175	[30]
PVA		0.006–0.032	12.2–36.2	[31]
PVDF		0.0008–0.0061	12.0–90.3	[32]
PVA/SPPO		0.007–0.013	111–181	[33]
CSM		0.0018–0.009	9.0–32.3	[34]
PVA		0.0077–0.0175	21.5–88.8	[35]
PVA		0.0147–0.0347	29.5–62.6	This research
